# Accurate Estimation of Nucleic Acids by Amplification Efficiency Dependent PCR

**DOI:** 10.1371/journal.pone.0042063

**Published:** 2012-08-17

**Authors:** Nilanjana Chatterjee, Tanmay Banerjee, Santanu Datta

**Affiliations:** Molecular Biology Section, AstraZeneca India Private Limited, Bangalore, Karnataka, India; University of Navarra, Spain

## Abstract

Accurate estimation of template - DNA or RNA by real time PCR is dependent on the amplification efficiency (F) of the reaction. The analytical equation describing the kinetics of PCR that is influenced by template re-annealing is formulated. It predicts the gradual reduction of F - from its initial value of 2, leading to template saturation. From an experimental standpoint, due to the exponential nature of the reaction a minute change in F can lead to a large error in the estimation of the initial template concentration. On the basis of individual variation in the amplification efficiency we have formulated a simple mathematical model and an MS Excel based data analysis software that allows accurate and automated quantification of initial template concentration. This method which does not require any normalisation with housekeeping genes was validated by transcript profiling of the genes in the TCA/glyoxylate cycle of *E. coli*. Consistent with published reports, we observed a precise and specific induction of the glyoxylate shunt genes when the bacteria was shifted from a six carbon glucose media to a two carbon source like acetate.

## Introduction

Real time PCR is a standard technique that is used to quantify in relative or absolute terms, the concentration of template - DNA or cDNA [1]–[5]. However, the precision of quantification technique depends on the mathematical model used for the evaluation [6]. Most of the currently available methodologies in real time PCR assume uniform/optimum amplification of all nucleic acid samples - DNA or cDNA fragments of different length and GC content. The Standard Curve Technique for absolute quantification assumes identical PCR amplification efficiencies for both the target template as well as the standard template DNA used for constructing the calibration curve [7]–[9]. While, the 2^ΔΔC^_q_ method used for relative quantification even assumes amplification efficiency, F, to be identical and optimum (i.e.  = 2) for both the target and reference templates [9]. A recently introduced standard curve independent methodology which involves an iterative simulation of the PCR process likewise estimates an optimal and common amplification efficiency for all samples in a group [10].

However in practice, F is rarely optimum or constant during the course of a PCR reaction. In fact amplification efficiency, F, varies from template to template and also for the same template during a PCR run between cycles. Due to the exponential nature of PCR even a small difference in the efficiency of individual amplification steps can have a significant effect on the amount of product accumulated after a limited number of cycles. Boggy and Woolf developed a two-parameter mass action kinetic model (MAK2) that can be fitted to qPCR data in order to quantify target concentration [11]. While, Guescini et al proposed a new method of nucleic acid quantification that does not require the assumption of equal reaction efficiency between unknowns and the standard curve [12].

Appreciating the problem that amplification efficiency could be less than 2 some investigators have also tried to estimate amplification efficiency by fitting the qPCR data to muti-parametric models using non linear regression [13]–[16]. However, the nature of bias of these analyses has also been pointed out. For example, the Sigmoidal Curve Fitting (SCF) method suffers from the inherent drawback of describing an amplification curve quantitatively even in the portion of the curve where the fluorescence signal has not risen above background and hence leads to biased estimates of amplification efficiency [17]. The alternate is to use statistical methods to evaluate the amplification efficiency but its robustness depends on the large number of experimental repeats [18],[19]. That there is a continuous decrease in amplification efficiency especially in the latter cycles has also been pointed out [20]. The idea to use the later region of the amplification curve which is not log-linear has also been attempted [21]. Lalam et al. [22] has focused on stochastic branching modeling assuming a saturation of amplification but has not taken self annealing of the amplified templates into consideration. While, Lievens et al. [23] has incorporated a complex phenomological cycle to cycle change in amplification efficiency but has not explained the mechanism of this change. In this work we have attempted to bring a simplified unity among the various alternative methods by estimating the amplification efficiency or amplification factor, F of a PCR reaction.

The proposed method does not involve any kind of simulation of PCR kinetics. Rather the exponential (log-linear) phase of each PCR reaction is actually determined by comparing ΔRn (background corrected normalized fluorescence intensity) values and the corresponding C_q_ (quantification cycle) values. Next, F is calculated in the linear range of individual PCR reaction and used directly for the estimation of the relative ratio of expression of the genes under study. This method of relative quantification is open ended and can be ubiquitously used with any detection chemistry available for real time quantitative PCRs. Due to its simplicity, we have chosen to use the non-specific double stranded DNA binding fluorescent dye SYBR Green I. However, to allow detection of only the specific product, fluorescence acquisition is done at a temperature greater than the Tm of primer dimers. Further, our proposed method does not require any specific internal controls like housekeeping genes or ribosomal RNA [25] to correct for individual and tube-to-tube variations in F provided we have a relatively accurate estimation of the number of input cells. In order to facilitate the efficient management and systematic evaluation of the large amount of data generated from each real time PCR experiment we have also developed a MS Excel based data analysis software, RARE (Real Amplification Ratio Extrapolation), using Macros. This Excel template (Anal_tool S1) along with input files(Data S1 & S2) are available as supporting information.

The proposed model is experimentally validated with respect to the well-known genes in the *E.coli* TCA cycle glyoxylate shunt pathway. It is well known that depending on the nature of the carbon source like acetate or glucose (C2 or C6), there is a significant change in metabolic flux distribution in the TCA cycle glyoxylate shunt pathway [26]–[32]. This is brought about by the induction of specific genes controlling the metabolic balance between these pathways. Using the mathematical model for relative quantification and the ensuing software we have done a comparative analysis of the transcript profiles of these genes with *E. coli* grown in acetate versus glucose as the sole carbon source. We could successfully monitor a precise and specific induction of the glyoxylate shunt genes over the TCA cycle genes in response to acetate as expected theoretically and thereby proving the validity of our method.

We have also theoretically probed into the mechanism of reduction of F which ultimately leads to template saturation. It is seen that template re-annealing is the major factor that leads to reduction in amplification efficiency.

## Methods

### Preparation of a SYBR Green I Containing 5X Buffer for Real Time Quantitative PCR

For all PCR applications a 5X PCR buffer containing optimum concentrations of SYBR Green I and ROX (Molecular Probes) was prepared. The latter is an internal passive reference dye that is used for normalization of fluorescence fluctuations in each sample. 10000X solution of SYBR Green I and ROX were obtained from Molecular Probes. The concentrations of the other components of the 5X PCR buffer such as Tris.Cl, MgCl_2_, KCl and DMSO (Sigma) were optimized following Taguchi’s principle [33](Text S1). As the optimum concentrations of these components vary with GC contents of the target template, we optimized three different 5X PCR buffers suitable for real time quantification of differential gene expression in different organisms (Figure S1 and Table S1). The GC content ranged from as low as *P. falciparum* (19.4% GC), moderate as *Escherichia coli* (50.8% GC) and high as M. *tuberculosis* (65.6% GC).

### Determination of the Dynamic Range of Quantification Provided by the SYBR Green Assay

In order to determine the range and resolution over which an accurate representation of the input amount of template is obtained with our methodology a quantitative PCR was done in duplicate with seven different dilutions (two fold) of a DNA template (PCR amplified and purified 386 bp region of *P. falciparum* IMPDH gene) starting from 1 pg/50 µl to 64 pg/50 µl. It was a four step PCR with the fourth step being set at 77°C, the Tm of primer dimers as determined from Figure S2(Text S1). The end products of all the seven duplicate PCR reactions were also subjected to a melting curve analysis to check the authenticity of the PCR products. Taq Polymerase used in PCR was procured from Finnzymes. The optimal buffer conditions were deduced using Taguchi optimization [33] (Table S1).

### Experimental Validation of the Proposed Model for Real Time RT-PCR Based Relative Quantification

Sixteen genes belonging to the *E. coli* TCA cycle/glyoxylate shunt pathway were considered for this study. We studied the differential expression of *E. coli* TCA cycle genes (citrate synthase, aconitate hydratase A, aconitate hydratase B, isocitrate dehydrogenase, malate dehydrogenase, 2-oxoglutarate dehydrogenase, dihydrolipoamide dehydrogenase, dihydrolipoamide succinyl transferase, succinyl CoA synthetase C, succinyl CoA synthetase D, succinate dehydrogenase and fumarate hydratase) as well as glyoxylate shunt genes (isocitrate dehydrogenase kinase, isocitrate lyase, malate syntahse A and malate synthase G) under altered carbon sources. For this *E. coli* was grown either in acetate or in glucose as sole carbon source and transcript profiling was done with SYBR Green I based real time RT-PCR with RNA isolated from cells grown under these two conditions.

*E.coli* strain JM101 was used for this experiment. A 15 ml culture of *E.coli* JM101 was grown in minimal media (containing per liter, 7 g of K_2_HPO_4_, 3 g of KH_2_PO_4_, 100 mg of MgSO_4_.7H_2_O along with 20 mM of NH_4_Cl as nitrogen source) supplemented with 22 mM glucose [34] and 1 µg/ml thiamine.HCl [35]. The inoculum was from an overnight culture of *E.coli* JM101 in LB which was washed twice in the minimal media to prevent carryover effect of rich LB media. To initiate a 15 ml culture of *E.coli* JM101 in the same minimal media supplemented with 44 mM sodium acetate [34] and 1 µg/ml thiamine.HCl [35], the *E. coli* strain was first subcultured thrice after inoculation from an LB agar plate for acclimatizing the organism in acetate, a C2 carbon source. Both the *E. coli* JM101 cultures were grown upto mid log phase at 37°C and then each culture was split into 3 sets each of volume 4 ml. Following this each fraction was treated with 10% (v/v) ice cold stop solution (10% buffer saturated phenol in ethanol) [36] to prevent degradation of mRNA. The cells were spun down at 3000 xg for 2 min, cell pellets were instantly flash frozen in dry ice and stored at −70°C until required. It is to be noted here that although both the cultures, glucose and acetate, were grown roughly upto mid log phase as determined by OD_600_ the acetate culture was very slow growing and never reached the exact OD_600_ at which the glucose grown *E. coli* culture was harvested. Hence to begin with there was a difference in cell number: we obtained approximately 5×10^9^ cells from 4 ml of glucose grown culture and roughly 3×10^9^ cells from 4 ml of acetate grown culture.

### Total RNA Extraction and Purification

Using the hot phenol method, total RNA was extracted from approximately 5×10^9^ cells obtained from each 4 ml set grown in 20 mM glucose and from 3×10^9^ cells obtained from each 4 ml set grown in 44 mM sodium acetate. The cell pellets from each set were resuspended in TE, pH = 8 and then equal volume of preheated water saturated phenol, pH∼7 was added. The cell lysates were gently mixed and then incubated at 64°C for 6 min with frequent mixing. The tubes were quickly chilled on ice and spun at 10,000 xg for 10 min at 4°C to collect the aqueous layer from each set. This was followed by another round of organic solvent extraction with equal volume of chloroform. The aqueous layer obtained from chloroform extraction was subjected to precipitation with 2.5 volumes ethanol and 300 mM NaOAc, pH 5.2. The (DNA + RNA) pellet obtained after 70% ethanol wash was resuspended in 100 µl of DEPC treated water.

For complete removal of genomic DNA contamination in the total RNA preparations each RNA sample was treated with 1 Unit of RNase free DNaseI (Roche)/µg of genomic DNA (1 cell is equivalent to 1 molecule of genomic DNA) in 1X DNaseI buffer, 0.1 M sodium acetate and 5 mM MgSO_4_ for 20 mins at 25°C. After DNaseI treatment the sample was extracted with phenol chloroform followed by ethanol precipitation.

Integrity of the total RNA preparations was checked by analysing these samples on a 1.2% agarose gel (Figure S4). The RNA samples were denatured in presence of 50% formamide, at 65°C for 5 min, before loading on gel. Clear and sharp 23S and 16S rRNA bands ensured intact total RNA isolation from both acetate and glucose grown cultures (Figure S4). Total RNA quality was also ensured from the A_260_/A_280_ ratio ≥1.9 and by monitoring the spectrum using a Beckman DU650 UV spectrophotometer. From the A_260_ values the amount of total RNA obtained from each 4 ml set of glucose or acetate culture was estimated.

### cDNA Synthesis by Reverse Transcription with Total RNA

Total RNA obtained per cell from glucose grown *E. coli* was three times more than that from the acetate culture. In order to take equal amount of total RNA (2 µg) from both the sets, glucose and acetate, for cDNA synthesis total RNA equivalent to 5×10^8^ cells was taken from each of the three sets of the acetate grown culture while from each of the three sets of glucose grown culture total RNA equivalent to 1.5×10^8^ cells was taken. To minimize tube to tube variation in cDNA synthesis, reverse transcription was carried out in a single tube format using a 100 ng/µl mixture of the sixteen gene specific reverse primers. The reverse transcription assay included 200 Units of SuperScript RNase H^−^ reverse transcriptase (Invitrogen), 500 µM dNTPs, 10 mM DTT, DNaseI treated total RNA (2 µg), 1.6 µl of 100 ng/µl reverse primer mix (so that 10 ng of each reverse primer is added) and First-Strand assay buffer (Invitrogen) at 1X final concentration in a 20 µl reaction. The cDNA synthesis assay as well as the sequence of addition of the different reactants was done following manufacturer’s instructions.

### SYBR Green I Based Real Time Quantitative RT-PCR

Sixteen TCA cycle/glyoxylate shunt gene specific primers (Table 1) which span roughly 300 bp regions towards the 3′ end of the respective *E. coli* genes and two pairs of intergenic primers (Figure S3) were designed using Primer3 software (Whitehead Institute for Biomedical Research). c-DNA synthesized from 2 µg of total RNA (equivalent to 5×10^8^ cells for acetate or 1.5×10^8^ cells for glucose) in each of the three sets of glucose or acetate grown cultures was split (@ 1 µl of cDNA per 50 µl PCR reaction) into 20 real time RT PCR reactions (16 PCR reactions with 16 pairs of *E. coli* gene specific primers and 4 control PCR reactions with 2 pairs of intergenic primers: two reactions per pair of intergenic primers - one with RNA template and the other with c-DNA template). Thus for each growth condition (acetate or glucose) a total of 60 RT-PCRs (20 reactions per set) were set up for 30 cycles with SYBR Green I containing PCR buffer optimised for moderate GC containing DNA templates (Table S1). Following PCR melting curve analysis was performed in the same instrument. Only after confirming that there was no amplification in the control reactions (which ensures absence of genomic DNA contamination in the RNA or cDNA samples) and also absence of non-specific products in the sample reactions data analysis was done using the software developed (RARE).

**Table 1:** Sequence of the 16 *E. coli* gene specific forward and reverse primers.

Primer Identifier	Gene Product	Primer Sequence
b0116F	dihydrolipoamide dehydrogenase	5′CGAAAGAAGACGGCATTTATGT3′
b0116R		5′TGTTTCTTACCGGCGATAACTT3′
b0118F	aconitate hydratase,acnB	5′TGGTTGTACCATCAAGCTGAAC3′
b0118R		5′GTCGATCTTCTCACCCTGTACC3′
b0720F	citrate synthase	5′TCACCGCGTGTACAAAAATTAC3′
b0720R		5′CTTCATACCGTCACTGTGCATT3′
b0721F	succinate dehydrogenase	5′AATCTGGACCTACAGACCATCC3′
b0721R		5′GCTTCGAATGTTTCTTCCAGAT3′
b0726F	2-oxoglutarate dehydrogenase,sucA	5′AGGTTTATTACGACCTGCTGGA3′
b0726R		5′CTGTTTCTGGTGAACGGACATA3′
b0727F	dihydrolipoamide succinyl transferase	5′TTCAACGAAGTCAACATGAAGC3′
b0727R		5′GACTGCCAGCTCTTTGATTTTC3′
b0728F	succinyl-CoA synthetase,sucC	5′CTGCACAGTGGGAACTGAACTA3′
b0728R		5′GGTACGTTAACACCCACTTCT3′
b1136F	isocitrate dehydrogenase	5′GGTTAAACACCCTGAACTGACC3′
b1136R		5′ACTTCATGATGTTGCCTTTGTG3′
b1611F	fumarate hydratase	5′GCGGTGGGTACTGGACTAAATA3′
b1611R		5′CACACTGTGTTGGGTTCACTTT3′
b2976F	malate synthase G	5′AAAATGGGCATTATGGATGAAG3′
b2976R		5′CTGTACATGTCTGCCATCAGGT3′
b4015F	isocitrate lyase	5′CTGAATGCCTTTGAACTGATGA3′
b4015R		5′AGTACGGAAGAAGCCTTCACTG3′
b4016F	Isocitrate dehydrogenase kinase phosphatase	5′AAGTCATTTACAGGTGGCGAAC3′
b4016R		5′GCAGCCGATAGCCATATACAAT3′
b4014F	Malate synthase A	5′GCAGGGTCAGGAAATCAATTA3′
b4014R		5′TACATCGAAGCGTGGATCTCT3′
b3236F	malate dehydrogenase	5′GAGAGAAGAAACGGGCGTACT3′
b3236R		5′AAGTTGAAGTGCCGGTTATTG3′
b0729F	succinyl-CoA synthetase,sucD	5′AACTGGCTT GGTAACGTGCT3′
b0729R		5′GGTGAATGCAAAATCGGTATC 3′
b1276F	aconitate hydratase.acnA	5′GACATAATGCAAAATGCCGTC3′
b1276R		5′CTTGGTATTCGTGTGGTGATTG3′

### Real Time Quantitative RT-PCR Data Analysis

The fluorescence data (ΔRn) and cycle number (n) values of each RT-PCR obtained from the Gene Amp 5700 Sequence Detection System (Perkin Elmer) were exported to the MS Excel based gene quantification software (RARE) for the automated estimation of the relative expression ratios. The program first determined the exponential phase of each PCR reaction (where N = N_0_F^n^ is valid; N_0_ is the initial template concentration while N is the final template concentration after n cycles of PCR), then calculated the amplification efficiency F for each gene in the log-linear range and the corresponding C_q_ (quantification cycle) values at a user defined ΔRn value which was selected to be 1 for all reactions followed by determination of an average of the individual F and C_q_ values of the three identical sets of RT-PCR reactions for a particular gene. These mean F and C_q_ values were subsequently used by the software to estimate the relative ratios under acetate and glucose with respect to the gene of minimum expression which has maximum F∧C_q_ value. The gene of minimum expression in glucose media was isocitrate dehydrogenase kinase while that in acetate media was fumarate hydratase. However, to compare between the two conditions the relative expression ratios under both glucose and acetate were calculated with respect to isocitrate dehydrogenase kinase, the gene of lowest expression in glucose.

At the time of calculating the relative expression ratios normalization was done with respect to total number of cells. This was in keeping that the total number of cells taken from the acetate grown culture was thrice the amount of cells taken from the glucose grown culture so the relative expression ratios of the transcripts in acetate medium were divided by a factor of 3.

## Results

### Mathematical Model for PCR Kinetics

The dynamics of the exponential synthesis of double stranded DNA during PCR is well understood. In contrast, there is a lack of clarity regarding the mechanisms that lower down amplification during the later cycles leading to template saturation, a phenomenon known as “plateau effect” [37]. It is thought that parameters like substrate depletion, inactivation of the polymerase enzyme, product inhibition and fractional re-annealing of the template strands, negatively influence the reaction which eventually leads to saturation. It can be seen from the following, that the gradual tapering of the amplification efficiency that ends in nearly complete cessation of synthesis can be derived analytically, resulting from the hybridization of the complementary templates.

In a PCR, the annealing of the primer to the template proceeds prior to chain elongation. During this step a fraction of the template also reassociates, thereby reducing its accessibility to the primer. If C_0_ is the total concentration of the template DNA, K is the second order rate constant and t is the time of annealing, then the concentration of single stranded template, C, remaining after template annealing is given by the following equation as defined by Wetmur and Davidson [38],


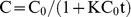
(I)

Since the available single stranded DNA is doubled during the PCR (assuming optimum annealing and extension), the total concentration of DNA produced after the first cycle would be
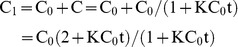
(II)Using similar argument the concentration of DNA after the second cycle of PCR would be
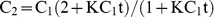
(III)Thus after n cycles of PCR the concentration of DNA would be given by
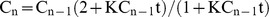
(IV)where Cn−1 is the concentration of DNA after n−1 cycles.

Using equations I to IV, the amount of DNA that is PCR amplified in each cycle can be calculated from the amount of DNA that was synthesized in the previous cycle. It is thus evident from this analysis that from the very beginning the amplification efficiency is not 2.0 but marginally less. In fact the amplification efficiency in any cycle is given by the relation,
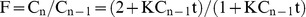
(V)F reaches the limit value of 2.0 when C_n−1_ t approaches zero.

**Figure 1:**
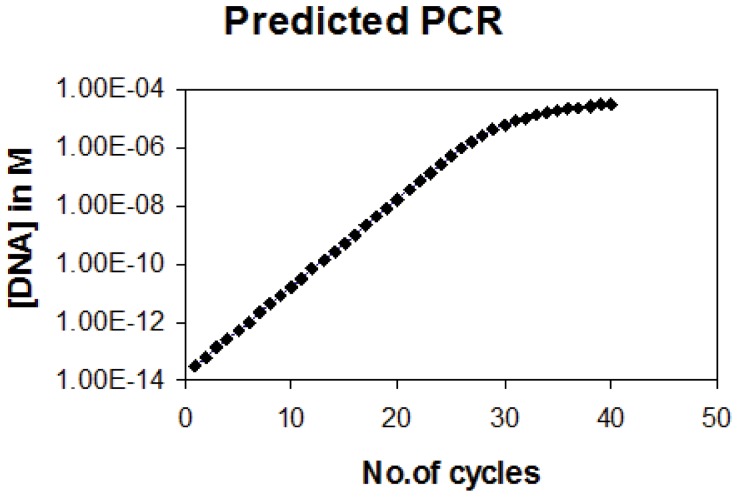
Theoretically predicted PCR profile with an initial template concentration of 1 pg/100 ul and fragment length of 1000 bp.

**Figure 2:**
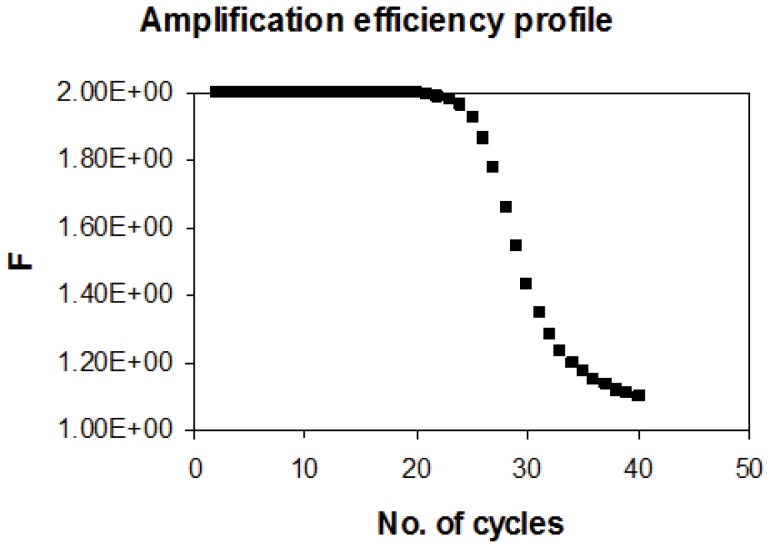
Theoretically predicted variation of amplification factor with number of cycles in an experiment where the initial template concentration is 1 pg/100 ul and length of the template 100 bp.

Using the equation of K = 3×10^5^L^0.5^/N mole^−1^ sec^−1^ as calculated by Wetmur and Davidson [38], the rate constant for a fragment of length 1000 bp is 10^4^ mole^−1^ sec^−1^. If we start with a PCR reaction of 1 pg in a 100 ul (33 fM) volume and use 30 sec as the annealing time, we can simulate a PCR. The data presented here (Figure 1) indicate that, as found experimentally the amplification saturates after 2−4×10^6^ fold amplification and the amplification efficiency (F) starts reducing significantly after 20 cycles (Figure 2). In this analysis other factors impacting amplification such as oligo annealing to the template and polymerase efficiency are assumed to be optimal. In case where it is non optimal, the initial value of F will be less than 2 but the phenomenon of reduction in F as depicted in Figure 2 will remain nearly the same. Due to this transition in PCR from exponential to quasi-linear and finally to a plateau phase, a dynamic change in the amplification efficiency is seen during the course of the reaction.

### Mathematical Model for Relative Quantification

Real time PCR ensures quantification in the exponential phase and is not affected by any reaction components becoming limited in the plateau phase [7], [39]. This however does not indicate that in the exponential phase PCR has an efficiency of 100% i.e. F = 2. In spite of this obvious caveat the most used real time PCR based quantification strategies such as the Standard Curve as well as the Comparative C_q_ methods assumes uniform/optimum amplification efficiency for all reactions. These methodologies are probably influenced by variations in amplification efficiency. To take into account this inherent variability (amplification efficiency) of the PCR technique, a simple mathematical model for relative quantification based on the calculation of amplification efficiency of individual PCR reaction is proposed here. This model is derived from the exponential equation N = N_0_ F^n^, describing PCR kinetics, where: N_0_ = initial amount of target template, N = amount of amplified product accumulated after n cycles and F = amplification efficiency which may vary from 1 to 2.

### Estimation of PCR Amplification Efficiency, F

In the SYBR Green I based real time quantitative PCR assay the fluorescence signal acquired at the end of each cycle is denoted by ΔRn, where ΔRn = (R_n_^+^)−(R_n_^−^). Here, R_n_^+^ is the normalised total fluorescence signal intensity, R_n_^−^ is the normalised background fluorescence and ΔRn is the background corrected normalised fluorescence intensity at any time. Now, as the amount of template goes on increasing from one cycle to another the fluorescence signal intensity also amplifies with the progress of the PCR reaction until saturation/Plateau phase is reached, so ΔRn is proportional to N in the exponential/log-linear phase.

Therefore, 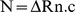 (where: c is an arbitrary constant).

Substituting N by ΔRn.c in N = N_0_ F^n^ we can write for a particular template,
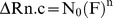
(1)


Taking log on both sides of equation **1** we arrived at the following equation:
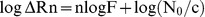
(2)


**Figure 3:**
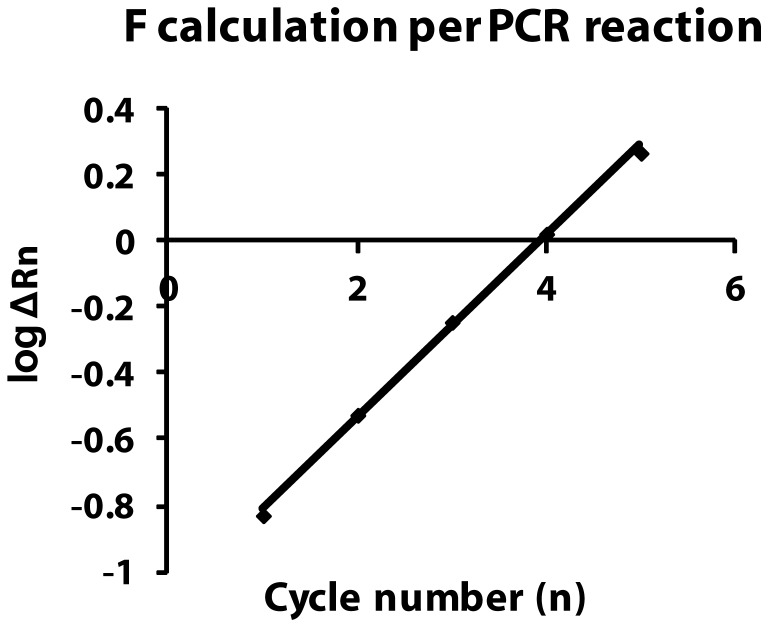
Graphical representation of the eqn. logΔRn = nlogF + log (N_0_/c). A straight line with a slope of logF and an intercept of N_0_/c on the Y-axis is obtained by plotting LogΔRn versus n. From the slope logF, the amplification efficiency of each individual PCR reaction can be calculated.

Equation 2 represents a straight line with a slope of logF and an intercept of logN_0_/c on the Y-axis that is obtained by plotting logΔRn versus n (Figure 3). From the slope logF, the amplification efficiency of each individual PCR reaction can be estimated.

### Relative Quantification Module

Unlike the 2^ΔΔC^_q_ method, our proposed model for relative quantification is independent of the assumption of constant/optimum amplification efficiency. Once the amplification efficiency of each PCR reaction is calculated from the corresponding reaction kinetics (using equation **2**), it is directly used for the estimation of the relative ratio of expression of the transcripts under study. Moreover, the computation of efficiency of individual amplification reaction makes it redundant to have an internal standard that is used for normalising the tube-to-tube variations in amplification efficiency provided a counted number of cells from a culture is used as input in the given experiment. Under this restriction the proposed model has the ability to perform relative comparisons without the use of internal controls like housekeeping genes or ribosomal RNA which are generally presumed to remain unaltered under most conditions. This is also not an appropriate assumption as in practice it is difficult to find a particular gene which can be unambiguously used as a reference. The model for relative quantification is derived from equation **1**. For two different genes or transcripts 1 and 2, equation **1** can be written as:
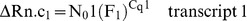
(3)

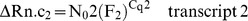
(4)where C_q_ is the cycle at which a threshold is reached and it is a fractional number.

At a particular value of ΔRn, comparing equations **3** and **4** we get the relative ratio of expression (N_0_1/N_0_2) of transcripts 1 and 2.
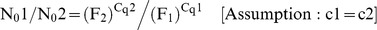
(5)


The assumption c_1_ =  c_2_ is valid among templates that do not have a large difference in their %GC content.

If there are ‘m’ target genes under study, then the ratio of relative levels of mRNA expression under a particular condition is given by:
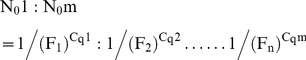
(6)where N_0_1, N_0_2,………., N_0_m represent the initial amounts of the m genes or transcripts under study at the beginning of the RT-PCR reaction. Furthermore, the relative ratio of expression is calculated with respect to the gene or transcript with minimum expression. So, if target gene 1 has minimum expression under a particular experimental condition then the ratio of relative levels of expression with respect to gene/transcript 1 is given by:



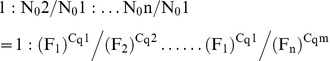
(7)(Note: gene of minimum expression will have maximum F^Cq^ value).

### Automated qPCR Data Analysis by RARE

**Figure 4:**
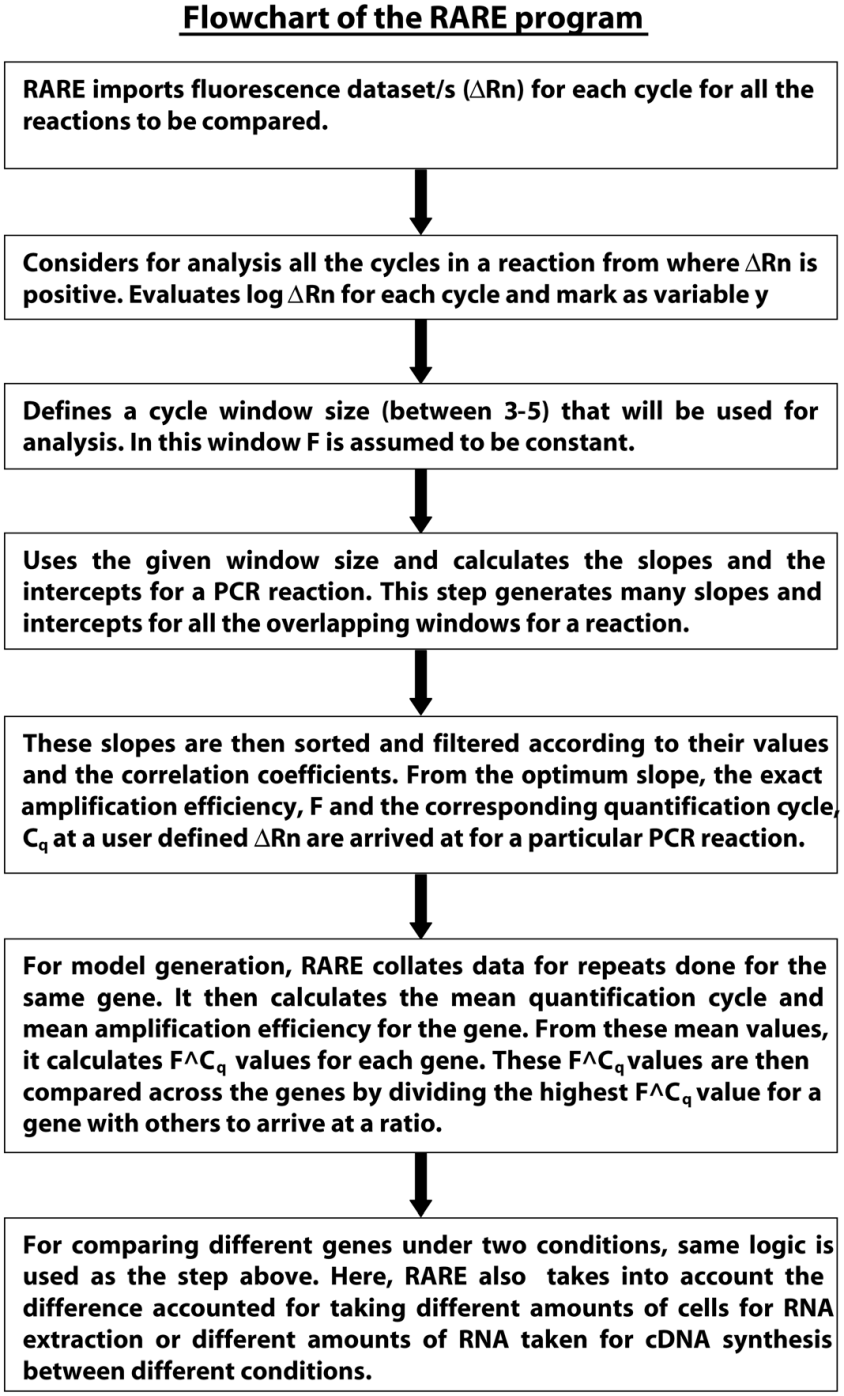
Flow chart of the analysis used in RARE.

RARE is an Excel based quantification tool to analyze qPCR data. Flow chart of the RARE program is depicted in Figure 4. Data analysis by RARE involves three major steps as follows.

1. Uploading of fluorescence intensity data from the PCR machine on to an EXCEL template.
2. Model creation using input data.
3. Determination of relative ratio within or across models.

Step 1: In this step, background subtracted fluorescence intensity data (ΔRn) from the instrument is loaded on to an EXCEL sheet. It is to be noted that background subtraction is done by the software that comes with the qPCR instrument. At present RARE works for Gene Amp 5700 Sequence Detection System (Perkin Elmer) but can be made to accept different input formats.

Step 2: Algorithm to create a model from the background subtracted fluorescence intensity data is based on the following sub steps.

- As input data for a target gene/transcript to be quantitated consists of cycle number (n) and corresponding background subtracted fluorescence intensity data (ΔRn) so RARE algorithm starts to look for a region, where ΔRn values for the reaction stays positive till the last cycle. This selected region of cycle number and fluorescence values will only be used for further analysis.
- In this step, RARE algorithm searches for the linear most sub-range window from the earlier selected range of values. This sub-range window can be defined by the user. For a 30 cycles PCR run, we have used 4 cycles to analyze the data. This sub-range window is used as an overlapping window length to check for linearity in the already found range. Linearity of the sub-range window is monitored by fitting first order linear equation between log_10_ΔRn & cycle number (represented by equation **2**). Tracking parameters like slope & correlation coefficient for each fitted sub-range window RARE algorithm selects the best sub-region to be used for quantification. As described by equation **2**, slope corresponds to amplification efficiency. Two filters are used to select the sub-range window:
  - Amplification efficiency between 1.5 and 2.0.
  - Correlation coefficient greater than 0.95.

If more than one sub-range window passes both the filters then the sub-range window starting with lower cycle number is selected for quantification. Gene is not quantified, in case none of the sub-range window passes through both the filters. Once the sub-range window is selected, slope and intercept on Y-axis for that sub-range window are used to determine the quantification cycle, C_q_, at a user defined ΔRn. This user defined ΔRn threshold is the same for all the genes to be quantified within the model. The quantitative factor, amplification efficiency to the power of quantification cycle, C_q_ (F∧C_q_), gives the value to compare with rest of the genes in the model. Mathematical model for the algorithm used in this step is described by equations **3**–**7**.

Step 3: Last step is used to compare genes within or across models. Here, the relative ratio of expression for a particular gene is determined by taking the ratio of highest quantitative factor (F∧C_q_) value of a gene within or across the models to the quantitative factor (F∧C_q_) value for that gene. Thus, the relative ratio for the gene with highest quantitative factor value within or across the model is one. In this step, if required, RARE also normalizes for differential amounts of total RNA used across models.

**Figure 5:**
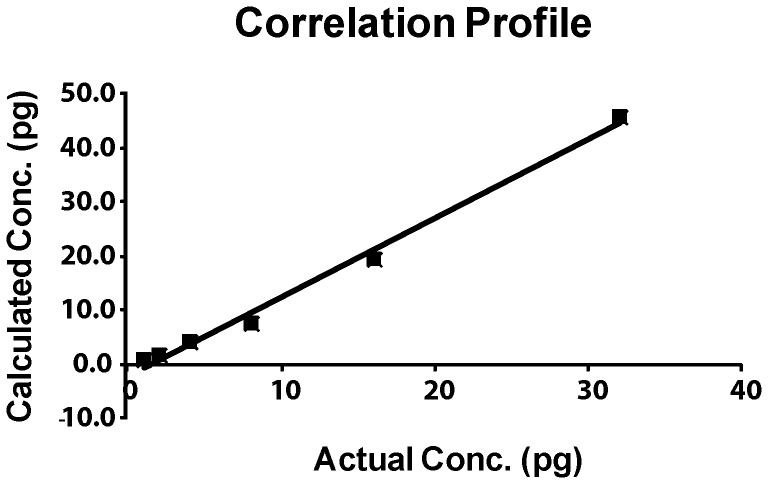
Correlation profile between the actual and the calculated initial template concentrations.

### Sensitivity and Dynamic Range of Quantification

PCR buffer conditions were optimized with respect to an AT rich *P. falciparum* DNA as well as a GC rich *M. tuberculosis* DNA so that the proposed model could be applied ubiquitously for the quantification of any DNA falling between these two extreme ranges (20–70% GC). We chose a 386 bp *P. falciparum* DNA for this study. Real time quantitative PCR was performed with seven different initial amounts of this 386 bp DNA template - ranging from 1 pg to 2, 4, 8, 16, 32 and 64 pg in duplicate to investigate the sensitivity and dynamic range of quantification provided by the SYBR Green I assay. Seven pairs of overlapping amplification plots were obtained (data not shown). These overlapping amplification plots were indicative of minimal tube-to-tube variations in the PCR.

**Table 2:** Relative levels of mRNA expression in TCA cycle/Glyoxylate Shunt pathway under acetate and glucose.

Gene No.	Gene product	F_cal_ (Acetate)	F_cal_ (Glucose)	Ace/Glu (F = 2) (A)	Ace/Glu (F_cal_) (B)	Ace/Glu (Oh et al data [40]) (C)
**b0720**	citrate synthase	1.92	1.93	1.95	1.96	1.79
**b1276**	aconitate hydratase, acnA	1.90	1.80	4.66	1.06	1.25
**b0118**	aconitate hydratase, acnB	1.95	1.96	1.26	1.32	2.17
**b1136**	isocitrate dehydrogenase	1.80	1.87	0.60	0.93	1.32
**b4016**	isocitrate dehydrogenase kinase	1.91	1.82	37.32	8.20	2.41
**b4015**	isocitrate lyase	1.84	1.92	10.55	15.67	3.39
**b2976**	malate synthase G	1.94	1.95	4.78	4.75	2.83
**b4014**	malate synthase A	1.94	1.83	18.07	4.75	-
**b3236**	malate dehydrogenase	1.87	1.94	4.67	6.47	1.73
**b0726**	2-oxoglutarate dehydrogenase	1.94	1.87	3.14	1.33	1.40
**b0116**	dihydrolipoamide dehydrogenase	1.92	1.84	0.26	0.13	1.13
**b0727**	dihydrolipoamide succinyl transferase	1.95	1.98	0.57	0.67	1.42
**b0728**	succinyl CoA synthetase,sucC	1.91	1.93	1.13	1.31	1.65
**b0729**	succinyl CoA synthetase,sucD	1.91	1.92	2.74	2.59	1.55
**b0721**	succinate dehydrogenase	1.99	1.90	2.28	0.85	1.41
**b1611**	fumarate hydratase	1.90	1.73	0.95	0.12	1.45

The relative ratios are calculated with respect to the gene of minimum expression. Correlation coefficients of relative ratio (acetate/glucose) calculated with F = 2 (column A) and F_cal_ (column B) with Oh et.al. data [40] (column C), are 0.46 and 0.85 respectively.

When quantification cycle, C_q_, was plotted against the input amount of target template, N_0_, at a particular ΔRn value ( = 1), a linear relationship was obtained suggestive of equal amplification efficiency among the diluted samples of the same gene for both the replicate series A and B. Indeed from the slopes (slope  = 1/logF) of the two ‘C_q_’ versus N_0_ plots, corresponding to the two duplicate series, the amplification efficiencies were found to be 1.88 and 1.87 for the two replicates (Figure S5). Moreover, the overlapping curves with a single peak obtained from melting curve analysis (data not shown) also indicate the selective detection of only the specific product. Next, by applying the relative quantification module (eqn. **7**) the ratio of the input amounts of the DNA template was estimated and it came to be 1: 1/1.85: 1/4.3: 1/7.7: 1/19.6: 1/45.9. When this calculated ratio was compared to the actual input ratio 1: 1/2: 1/4: 1/8: 1/16: 1/32, it was observed that the calculated ratio matched well with the actual input ratio upto 32 pg (Figure 5). The results indicate an overall correlation coefficient of 0.99 between the predicted and the actual initial template concentrations. However, it was seen that beyond the input template concentration of 32 pg the accuracy of the dynamic range started deviating. This was probably driven by limited cycles to reach saturation. At an input concentration of 64 pg and above it would require 14–15 cycles to reach saturation concentration of 1–2 ug depending on the amplification efficiency. Taking template re-annealing in the later cycles into consideration it would seem that there are a limited no of cycles available at this concentration where F remains constant and thereby causing the deviation.

### Experimental Validation of the Relative Quantification Model

**Figure 6:**
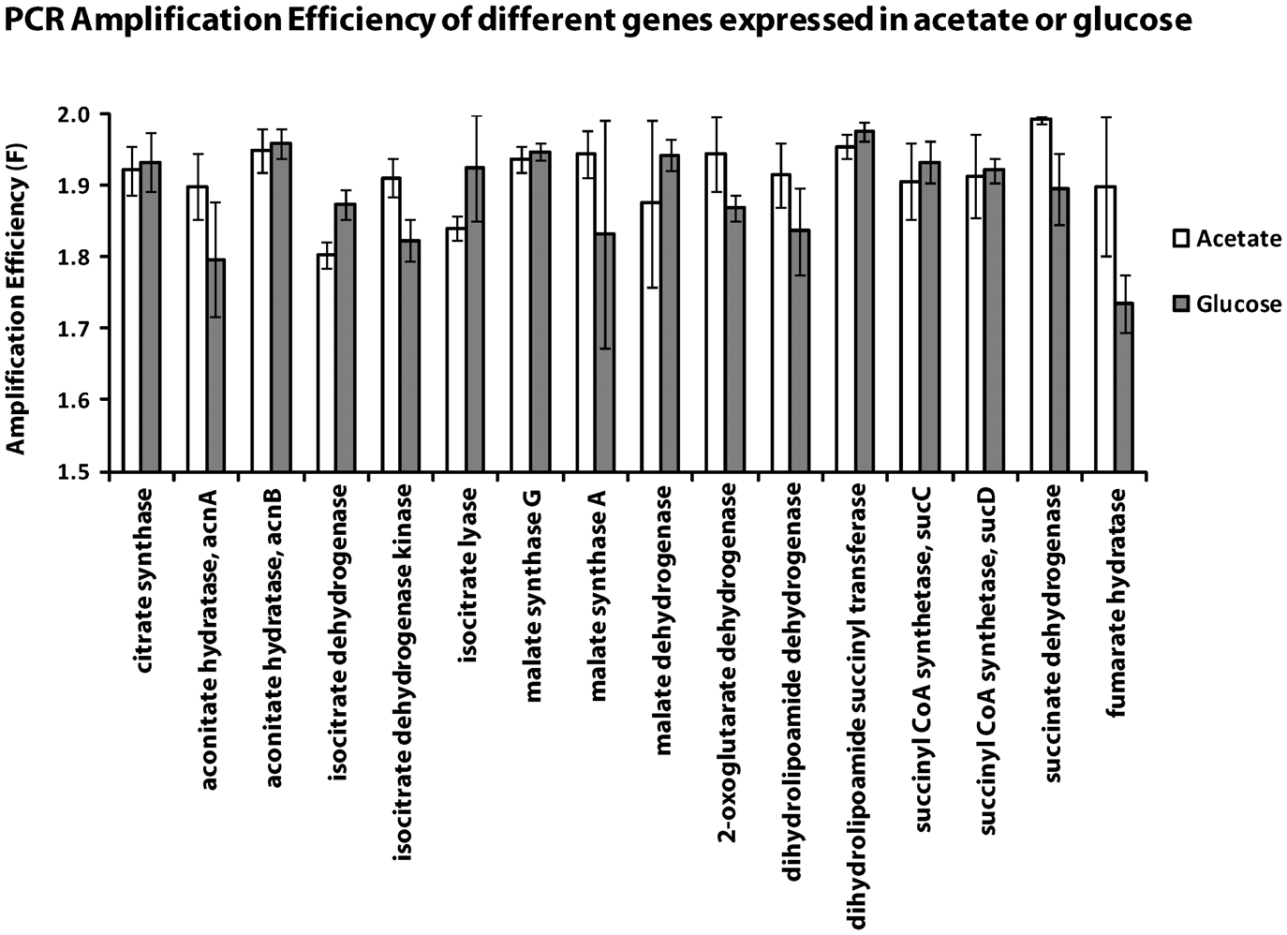
Amplification efficiency determined by RARE for TCA cycle/Glyoxylate Shunt genes expressed under alternate carbon sources. Mean amplification efficiency and standard deviation were calculated from three identical PCR reactions for either acetate (white bar) or glucose (grey bar) and plotted against the different genes under study.

The relative quantification model was experimentally validated with the sixteen *E. coli* TCA cycle/Glyoxylate Shunt genes. These genes along with the list of the forward and the reverse primers are described in Table 1. We chose the TCA cycle/Glyoxylate Shunt pathway as a model pathway for experimental validation as it is well characterised in *E. coli* and there is significant information on the metabolic flux distribution in this pathway under altered carbon sources (C2 and C6) [26]–[32]. Metabolic flux distribution studies have revealed that the Glyoxylate Shunt genes for isocitrate lyase and malate synthase along with other acetate metabolizing enzymes that are induced in acetate are typically down regulated in the presence of glucose [30], [32]. Here we have measured, with SYBR Green I based real time RT-PCR, the relative expression levels of the sixteen mRNAs of this pathway when *E. coli* was grown separately under glucose and acetate as the sole carbon source. The quality control of RNA is an important parameter to check and this is indicated by the integrity of the ribosomal RNA (Figure S4). A comparative analysis of the transcript profiles obtained under these altered physiological conditions (acetate versus glucose) revealed upregulation of isocitrate lyase, malate synthase and isocitrate dehydrogenase kinase (Table 2, column B) in acetate medium, the last one being the key regulatory enzyme at the TCA cycle/Glyoxylate Shunt branch point. In glucose medium, normal TCA cycle operates and the glyoxylate shunt pathway is switched off. Accordingly, we observed minimum expression of the branch point regulatory enzyme isocitrate dehydrogenase kinase in glucose medium (Table S2, column B). The expression of genes in the two media was compared with the published microarray expression profiles [40]. We have calculated the relative ratios with F = 2 (Table 2, column A) and F = F_Cal_ as evaluated by our method (Figure 6 and Table 2, column B). The correlation coefficients of relative ratios (acetate/glucose) with the Oh et al. microarray data [40] (Table 2, column C) was 0.46 with F = 2 compared to 0.85 with the use of F_Cal_, thus indicating the superiority of our method.

## Discussion

It has been often appreciated that the amplification efficiency is a very important variable in qPCR data analysis. In fact a mere 5% change in amplification efficiency from optimum would produce a massive 46 fold reduction in the quantity of product in a 30 cycle reaction. This inherent nature of PCR is troublesome when one is attempting to measure the initial concentration of the template. We have formulated the analytical equation that describes PCR. It clearly shows that in the initial cycles the amplification efficiency is constant and due to the phenomenon of template re-annealing the amplification efficiency is drastically reduced in the later cycles. Based on this analytical understanding of the internal dynamics of PCR we have developed a simple mathematical model that back calculates the initial concentration of the template by using the amplification efficiency of each PCR reaction during the early phase. In addition we have described through phenomenological equations why the amplification efficiency, F, reduces from its optimum value of 2. It has been appreciated for some time now that the PCR fluorescence data contains information about amplification efficiency and computing efficiency from the kinetics of individual PCR reactions is theoretically possible [41]. Some investigators attempted to estimate the amplification efficiency by fitting qPCR data using linear regression analysis [42], [43] while some others applied multi-parametric model fitting by non-linear regression [13]–[16]. These formulations were re-analysed by Zhao and Fernald who in addition used an algorithm that is resistant to the noise that arise out of fluorescence detection [16]. The use of data from early phase of the reaction where the amplification efficiency is maximum for a particular amplification reaction by our method overcomes these issues of fluorescence noise detection. It is also possible that fluorescence noise is amplified by the re-annealing of templates in the later phase. By theoretical modeling of PCR we have observed that template re-annealing is the primary reason for the continuous decrease in the amplification efficiency especially during the later cycles. It is the early cycles where amplification efficiency remains constant and our methodology helps in identifying this zone of constancy in F which is then used for calculating the initial template concentration. Further due to the logical build-in restriction in RARE, F calculated by this method, unlike by other methods [24], is never greater than 2 (Figure 6). Moreover, quantification in the log-linear range by our method and selective usage of fluorescence signal from the specific PCR product further contribute to amplification efficiency from not exceeding 2.

Another major advantage of this technique is the avoidance of using internal controls and having the possibility of simultaneously evaluating the expression profiles of a large number of genes. However this feature is valid in case where we have an accurate estimation of the number of input cells from where the mRNA is isolated. In most qPCR experiments the input tissue is less homogeneous and reference genes are still required to correct for variability in input quantity and composition. We have successfully validated this protocol and evaluated the modulation of the expression of the genes in the TCA cycle/Glyoxylate pathway due to nutritional shift from glucose to acetate. It is clearly seen that as expected the genes for the *aceBAK* operon in which the genes isocitrate lyase, malate synthase and isocitrate dehydrogenase kinase are induced during this shift (Table 2, column B) thus validating our proposed methodology of quantification of template DNA. The transcript profile of glucose and acetate grown *E. coli* has recently been investigated by microarray analysis [40]. Our results (Table 2, column B) are consistent with this data (Table 2, column C) compared to the use of the standard 2^ΔΔC^_q_ where F = 2 (Table 2, column A). It is often a practice to validate microarray data with qPCR. In the present study we have apparently attempted the opposite. This is because the upregulation of the glyoxylate shunt (described by the operon *aceBAK*) in *E.coli* when the carbon source shifts from glucose to acetate is one of the most thoroughly investigated and protein and metabolite levels have been monitored [26]–[32]. It might be interesting to note that the specific genes in this pathway namely *aceK* (b4016: isocitrate dehydrogenase kinase), *aceA* (b4014, b2976: malate synthase) and *aceB* (b4015: isocitrate lyase) are significantly upregulated as expected and more than the microarray data [40] seems to suggest. The upregulation values are in line with earlier metabolite profiling data.

The software that we have developed for automated data analysis with real time PCR provides the user with options of estimation of the amplification efficiency of each PCR reaction followed by computation of the relative fold change of expression of individual templates in a 96 well format. Moreover, it is open ended and can be suitably used with any commercial instrument capable of capturing the intensity of fluorescence at the end of each cycle.

## Supporting Information

Text S1 **Supplemental Data.** PCR buffer conditions for AT/GC rich DNA & four step PCR strategy for SYBR Green detection.
(DOCX)

Figure S1 Optimisation of SYBR Green I based PCR conditions.
(TIF)

Figure S2 Four step qPCR strategy with SYBR Green I.
(TIF)

Figure S3 Intergenic primers designed to check genomic DNA contamination in total RNA or cDNA preparations.
(TIF)

Figure S4 Integrity analysis of total RNA isolated from the *E.coli* cultures in glucose and acetate.
(TIF)

Figure S5 Determination of PCR amplification efficiency with seven different initial concentrations of a 386 bp DNA template.
(TIF)

Table S1 Optimised buffer conditions used for *P. falciparum*, *E. coli* and *M. tuberculosis* PCRs.
(DOCX)

Table S2 Relative ratios of TCA cycle/Glyoxylate Shunt genes.
(DOCX)

Data S1 **B222A.** RT-PCR reactions carried out under acetate as carbon source.
(TXT)

Data S2 **B111G.** RT-PCR reactions carried out under glucose as carbon source.
(TXT)

Tool S1 **RARE.** MS-EXCEL based analysis tools.
(XLSM)
