# Supplementary material for: Accurate Estimation of Nucleic Acids by Amplification Efficiency Dependent PCR
Source: PLoS One. 2012 Aug 17;7(8):e42063. doi: 10.1371/journal.pone.0042063 (PMC3422235; doi:10.1371/journal.pone.0042063)
Supplement: Text S1 — Supplemental Data. PCR buffer conditions for AT/GC rich DNA & four step PCR strategy for SYBR Green detection. (DOCX) [file pone.0042063.s010.docx]

# Supplemental Data

## Optimization of SYBR Green I based PCR conditions

In order to use SYBR Green I at a maximum (to get an intense signal) but PCR non-inhibitory concentration, optimization of SYBR Green I concentration was done with respect to an AT rich *P. falciparum* DNA as well as a GC rich *M. tuberculosis* DNA so that the proposed model could be applied ubiquitously for the quantification of any DNA falling between these two extreme ranges (20-70% GC). For the AT rich *P. falciparum* DNA template a final concentration of 0.7X (Figure S1A) while for the GC rich *M. tuberculosis* DNA template a final concentration of 1.5X (Figure S1B) of SYBR Green I was found to be optimum for use in 1X PCR buffer.

In order to minimise formation of non-specific products and primer dimers the concentrations of the different components, such as, Tris.Cl, MgCl_2_, KCl and DMSO in the SYBR Green I PCR buffer were optimized by measuring their effects on the amplification efficiency, yield and specificity of the PCR product (17)**.** Moreover, as PCR amplification efficiency not only depends on priming efficiencies of the primers but also on the melting behaviour of the amplicon so in all the optimisation PCRs with the GC rich *M. tuberculosis* genomic DNA we used a pre-melted template. As a quality control measure, the interference of the PCR additives, like DMSO and betaine, used in the optimized PCR buffers with SYBR Green fluorescence emission was also tested. Fluorescence emission (in appropriate fluorescence units) was found to increase with increasing DMSO concentration from 0% to 2%. With further increase in DMSO concentration (checked upto 4%) a gradual decrease in the signal intensity was observed. However, SYBR Green I fluorescence emission was found to be unaffected by the use of betaine at a final concentration of ~0.7M in 1X PCR buffer (data not shown).

From these rigorous optimization experiments three different sets of optimum buffer conditions for PCR with the three different templates (used in this study) of varying AT-GC content were arrived at ( Table S1).

## Designing of a four step PCR to prevent the detection of primer dimmers along with the specific product by SYBR Green I

# In order to overcome the major drawback of SYBR Green I chemistry of simultaneous detection of specific and non-specific products like primer dimers we devised a four step PCR (denaturation, annealing, extension and fluorescence acquisition) instead of the regular three step PCR (denaturation, annealing and extension) where the fourth step is set at a temperature 0˚ to 3˚C greater than the Tm of primer dimers (Figure S2B). Since the Tm of the primer dimers is significantly less than the Tm of the specific product, fluorescence signal acquisition at the fourth step will be solely from the specific product. Specific fluorescence monitoring was achieved by setting the fourth step of every cycle as the fluorescence acquisition step in the Gene Amp 5700 Sequence Detection System (Perkin Elmer). The melting temperature (Tm) of primer dimers required for setting up of a four step PCR is determined from melting curve analysis in the same instrument.

Melting curve analysis following standard three step PCR shows peaks corresponding to primer dimers as well as the amplified product. This is exemplified in Figure S2A which shows the melting profile of the 386 bp product amplified from *P. falciparum* genomic DNA with IMPDH gene specific primers by regular 3 step PCR. The difference in Tm of the primer dimers and the amplified product is about 6˚C. On the basis of this melting curve analysis when a four step PCR was done with the fourth step being set at 77˚C it ensured only the specific detection of the 386 bp product.

**Figure S1: Optimisation of SYBR Green I based PCR conditions**

**A.** PCR with 10 different concentrations (0.1X, 0.2X, 0.3X, 0.4X, 0.5X, 0.6X, 0.7X, 0.8x, 0.9X, 1X) of SYBR Green I (from lanes 3 to 12) to check upto what concentration amplification is not inhibited in the case of an AT rich 386 bp DNA (*P. falciparum*). Lane1, 400 bp DNA Marker; Lane2, PCR without SYBR Green I; Lane13, no template control. **(a)** Amplification was obtained upto 0.7X SYBR Green I (faint band in lane# 9) at the end of 20 cycles. **(b)** At the end of 25 cycles. **(c)** Amplification obtained at the end of 30 cycles. **B.** PCR with 15 different concentrations (0.1X to 1.5X) of SYBR Green I using a GC rich template (*M. tuberculosis*). Lane1, 100 bp DNA Ladder; Lanes 2-16, 0.1X to 1.5X of SYBR Green I. PCR was not inhibited even upto 1.5X SYBR Green I for a GC rich template.

**Figure S2: Four step qPCR strategy with SYBR Green I**

**A.** Determination of the Tms of the 386 bp specific product and primer dimers by melting curve analysis. Tm of primer dimers produced in the PCR with *P. falciparum* DNA was 77˚C while the Tm of the 386 bp specific product was 83˚C. **B.** 4 step PCR strategy versus standard 3 step PCR.

**Figure S3: Intergenic primers designed to check genomic DNA contamination in total RNA or cDNA preparations**

Two pairs of intergenic primers: LAC forward and reverse primers span an intergenic region in the Lactose operon of *E. coli* and TRP forward and reverse primer sequences correspond to an intergenic region in the Tryptophan operon of *E. coli*.

**Figure S4: Integrity analysis of total RNA isolated from the *E.coli* cultures in glucose and acetate**

The total RNA isolated from *E.coli* grown in glucose and acetate media were denatured by heating in the presence of formamide and were analysed by 1.2% denaturing agarose gel stained with ethidium bromide. M, rabbit muscle RNA; Lane 1, total RNA (acetate); Lane 2, total RNA (glucose). Both the RNA samples (lanes 1 & 2) have sharp 23S and 16S ribosomal RNA bands clearly indicating that the isolated RNAs are completely intact. Inclusion of the control RNA (lane M) on the gel allowed size determination of the ribosomal RNA bands and also ensured that the gel was run properly.

**Figure S5. Determination of PCR amplification efficiency with** **seven** **different initial concentrations of a 386 bp DNA template**

Quantification cycle, C_q_ versus N_0_ plot gives a straight line represented by the equation C_q_ = logN/logF -logN_0_/logF. This straight line has a negative slope (= 1/logF) and a positive intercept (= logN/logF) on the Y-axis. The equations: y = -1.5968Ln(x) + 16.013 and y = -1.5746Ln(x) + 15.909 represent the two straight lines obtained by plotting C_q_ versus N_0_ at ΔRn=1 for the two duplicate series: series A (solid triangle) & series B (open square) respectively. From the slopes of these two straight lines amplification efficiencies of the two replicates were determined to be 1.87 and 1.88.

# Data analysis using RARE:

We have provided MS-EXCEL based analysis tools named “RARE” (File name : swRARE.xlsm) for users. There are two input files:

1. B222A.txt (RT-PCR reactions carried out under acetate as carbon source)
2. B111G.txt (RT-PCR reactions carried out under glucose as carbon source)

One can use these files to analyze data as described in the manuscript.
