# Supplementary figures and images for: Accurate Estimation of Nucleic Acids by Amplification Efficiency Dependent PCR
Source: PLoS One. 2012 Aug 17;7(8):e42063. doi: 10.1371/journal.pone.0042063 (PMC3422235; doi:10.1371/journal.pone.0042063)

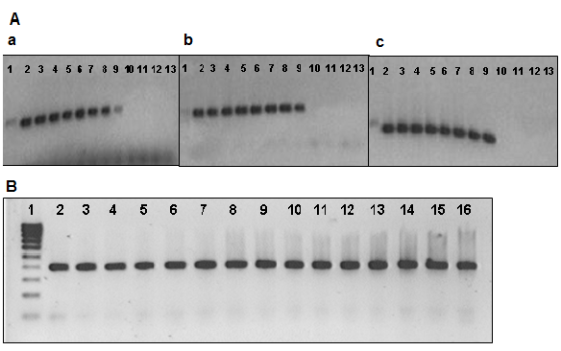

Supplement: Figure S1 — Optimisation of SYBR Green I based PCR conditions. (TIF) [file pone.0042063.s001.tif]

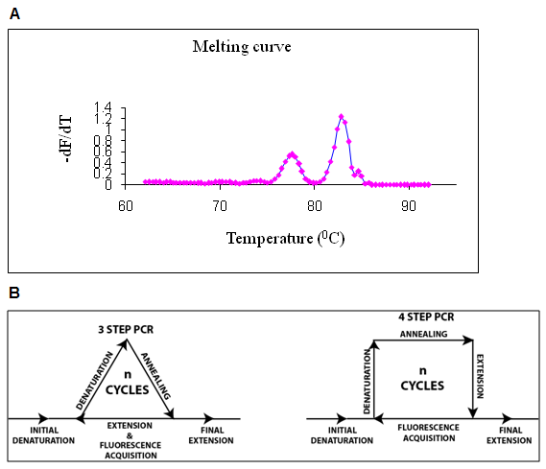

Supplement: Figure S2 — Four step qPCR strategy with SYBR Green I. (TIF) [file pone.0042063.s002.tif]

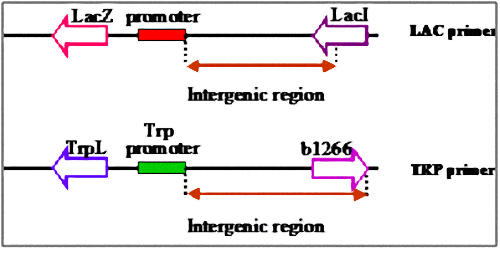

Supplement: Figure S3 — Intergenic primers designed to check genomic DNA contamination in total RNA or cDNA preparations. (TIF) [file pone.0042063.s003.tif]

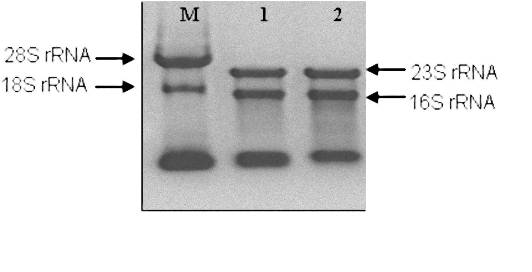

Supplement: Figure S4 — Integrity analysis of total RNA isolated from the E.coli cultures in glucose and acetate. (TIF) [file pone.0042063.s004.tif]

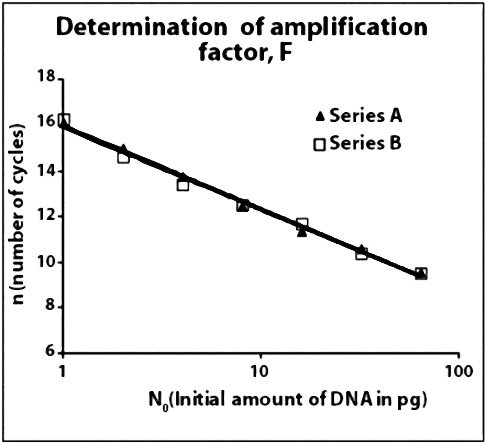

Supplement: Figure S5 — Determination of PCR amplification efficiency with seven different initial concentrations of a 386 bp DNA template. (TIF) [file pone.0042063.s005.tif]
