# Supplementary material for: Accurate Estimation of Nucleic Acids by Amplification Efficiency Dependent PCR
Source: PLoS One. 2012 Aug 17;7(8):e42063. doi: 10.1371/journal.pone.0042063 (PMC3422235; doi:10.1371/journal.pone.0042063)
Supplement: Table S1 — Optimised buffer conditions used for P. falciparum, E. coli and M. tuberculosis PCRs. (DOCX) [file pone.0042063.s006.docx]

**Table S1**

| **1X PCR Buffer Composition** | | | | | | | |
| --- | --- | --- | --- | --- | --- | --- | --- |
| **Organism** | **AT/GC content** | **10 mM Tris.Cl,** | **MgCl_2_** | **KCl** | **DMSO** | **Betaine** | **SYBR Green I** |
| *P. falciparum* | AT rich (19.4% GC) | pH = 8.1 | 3 mM | 50 mM | - | - | 0.7X |
| *E. coli* | Moderate AT/GC (50.8% GC) | pH = 8.8 | 1.5 mM | 50 mM | 4% | - | 1.5X |
| *M. tuberculosis* | GC rich (65.6% GC) | pH = 8.8 | 2.25 mM | 50 mM | 4% | - | 1.5X |
|  |  | pH = 8.8 | 2.25 mM | 50 mM | 2% | 0.7 M | 1.5X |

**Table S1: Optimised buffer conditions used for *P. falciparum*, *E. coli* and *M. tuberculosis* PCRs**
