# Supplementary material for: Accurate Estimation of Nucleic Acids by Amplification Efficiency Dependent PCR
Source: PLoS One. 2012 Aug 17;7(8):e42063. doi: 10.1371/journal.pone.0042063 (PMC3422235; doi:10.1371/journal.pone.0042063)
Supplement: Table S2 — Relative ratios of TCA cycle/Glyoxylate Shunt genes. (DOCX) [file pone.0042063.s007.docx]

Table S2

| **Gene No.** | **Gene product** | **Normalized Relative Ratio**  **(Acetate)**  **(A)** | **Normalized Relative Ratio**  **(Glucose)**  **(B)** |
| --- | --- | --- | --- |
| **b0720** | citrate synthase | 53.20 | 27.21 |
| **b1276** | aconitate hydratase | 5.85 | 5.53 |
| **b0118** | aconitate hydratase | 44.38 | 33.51 |
| **b1136** | isocitrate dehydrogenase | 789.82 | 845.17 |
| **b4016** | isocitrate dehydrogenase kinase | 8.20 | 1.00 |
| **b4015** | isocitrate lyase | 516.33 | 32.95 |
| **b2976** | malate synthase G | 17.86 | 3.76 |
| **b4014** | malate synthase A | 159.33 | 33.55 |
| **b3236** | malate dehydrogenase | 702.18 | 108.52 |
| **b0726** | 2-oxoglutarate dehydrogenase | 32.45 | 24.37 |
| **b0116** | dihydrolipoamide dehydrogenase | 18.47 | 145.05 |
| **b0727** | dihydrolipoamide succinyl transferase | 31.92 | 47.41 |
| **b0728** | succinyl CoA synthetase | 108.10 | 82.76 |
| **b0729** | succinyl CoA synthetase | 112.91 | 43.52 |
| **b0721** | succinate dehydrogenase | 15.18 | 17.75 |
| **b1611** | fumarate hydratase | 3.24 | 26.92 |

**Table S2: Relative ratios of TCA cycle/ Glyoxylate Shunt genes**

Relative ratios of mRNA expression under acetate (column A) and glucose (column B). The relative ratios are calculated with respect to isocitrate dehydrogenase kinase, the gene of minimum expression in glucose medium.
